# Supplementary figures and images for: Chemotype classification and biomarker screening of male Eucommia ulmoides Oliv. flower core collections using UPLC-QTOF/MS-based non-targeted metabolomics
Source: PeerJ. 2020 Aug 21;8:e9786. doi: 10.7717/peerj.9786 (PMC7444510; doi:10.7717/peerj.9786)

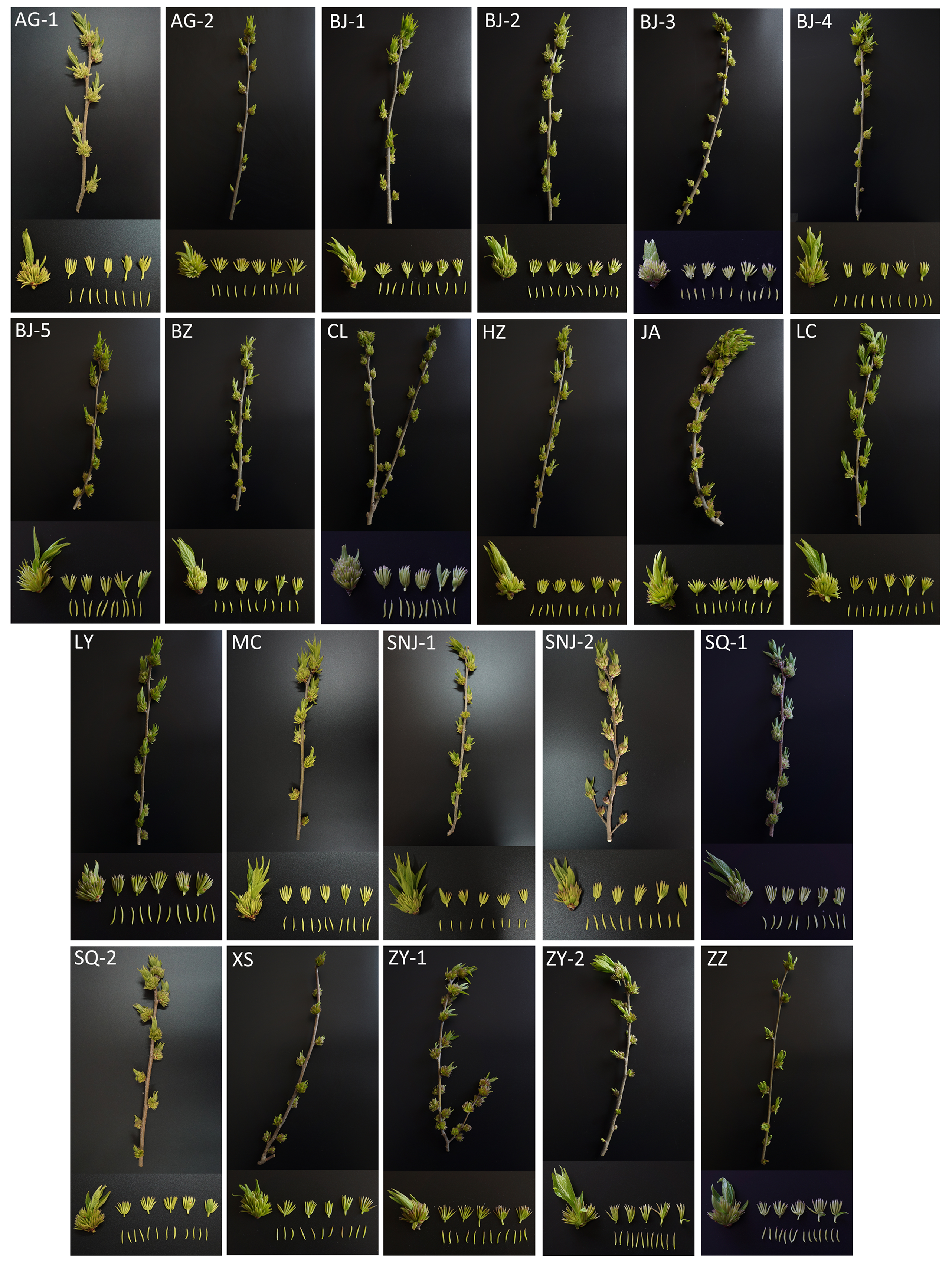

Supplement: Supplemental Information 1 [file peerj-08-9786-s001.png]

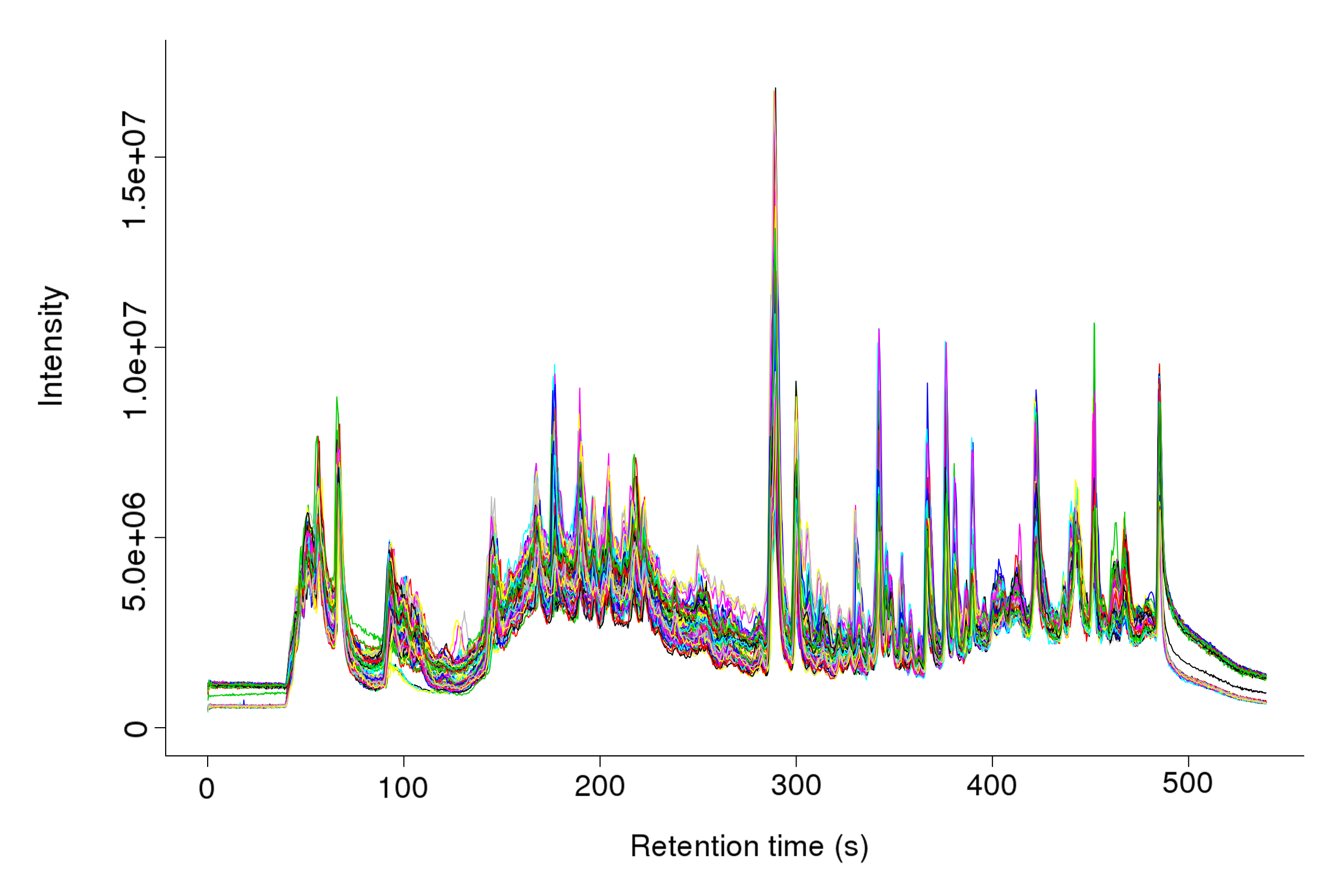

Supplement: Supplemental Information 2 [file peerj-08-9786-s002.png]

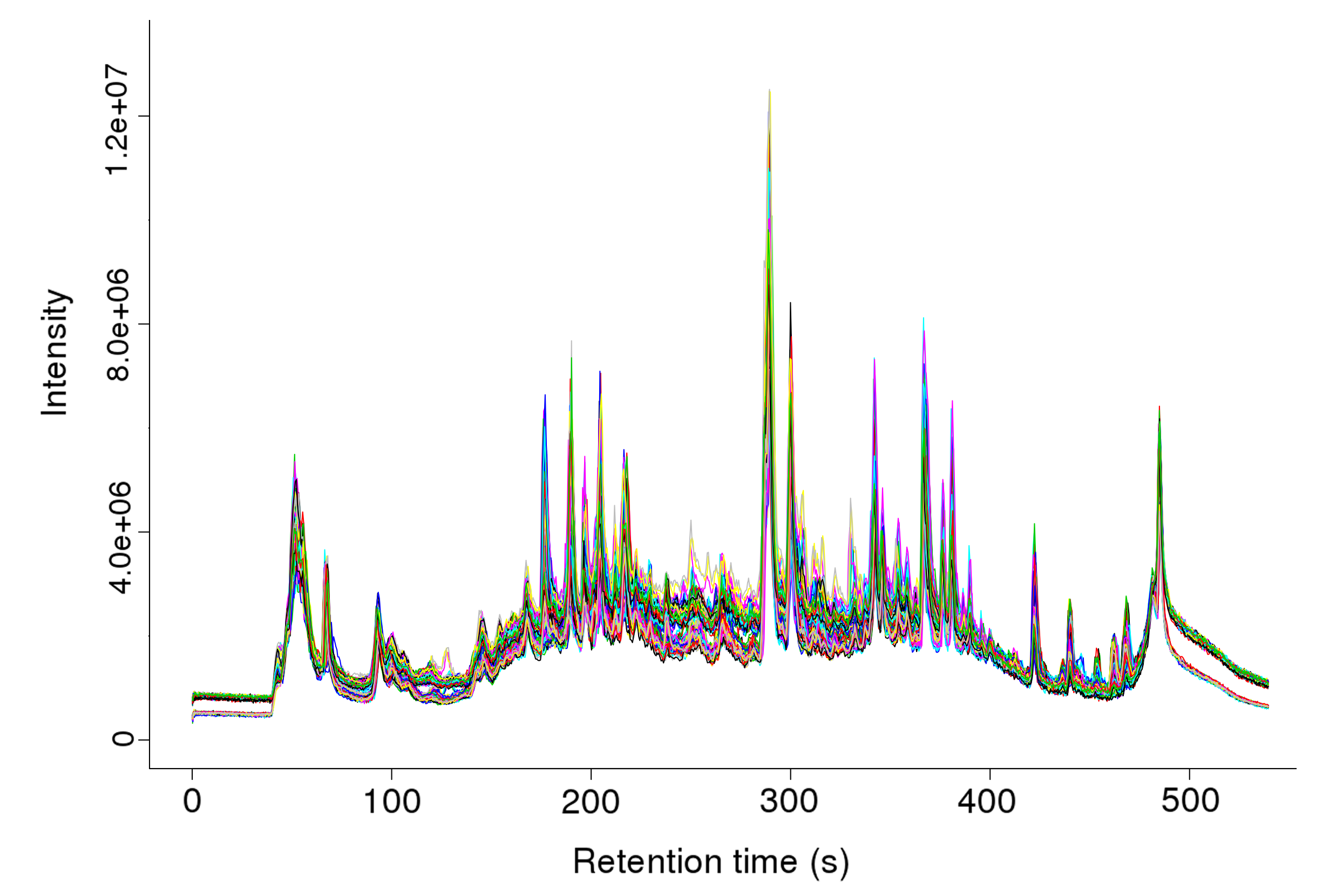

Supplement: Supplemental Information 3 [file peerj-08-9786-s003.png]

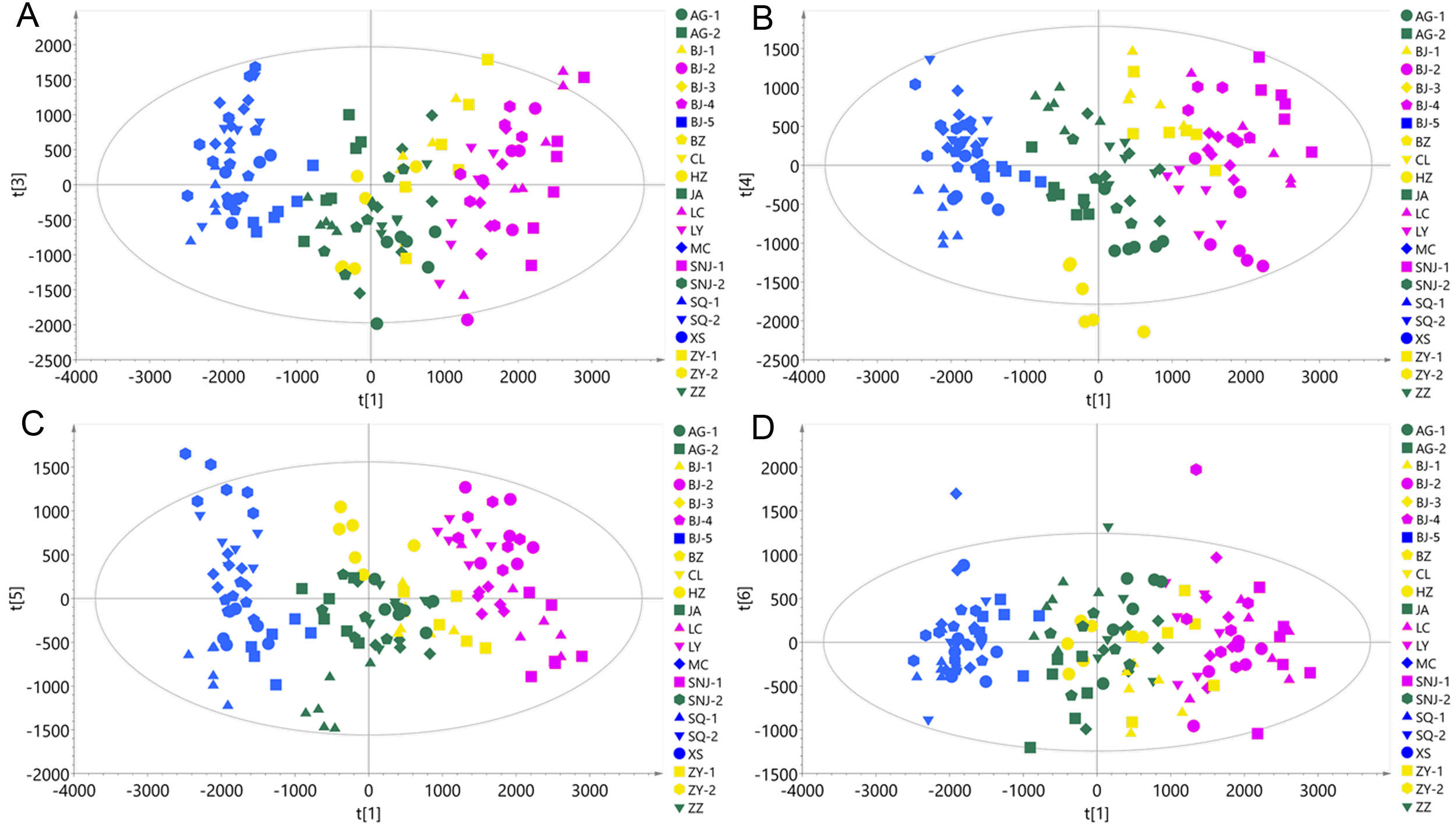

Supplement: Supplemental Information 4 — (A) PC1 pairing with PC3, PC3 accounting for 7.35% of the total variance; (B) PC1 pairing with PC4, PC4 accounting for 6.04% of the total variance; (C) PC1 pairing with PC5, PC5 accounting for 4.61% of the total variance; (D) PC1 pairing with PC6, PC6 accounting for 2.92% of the total variance. [file peerj-08-9786-s004.png]

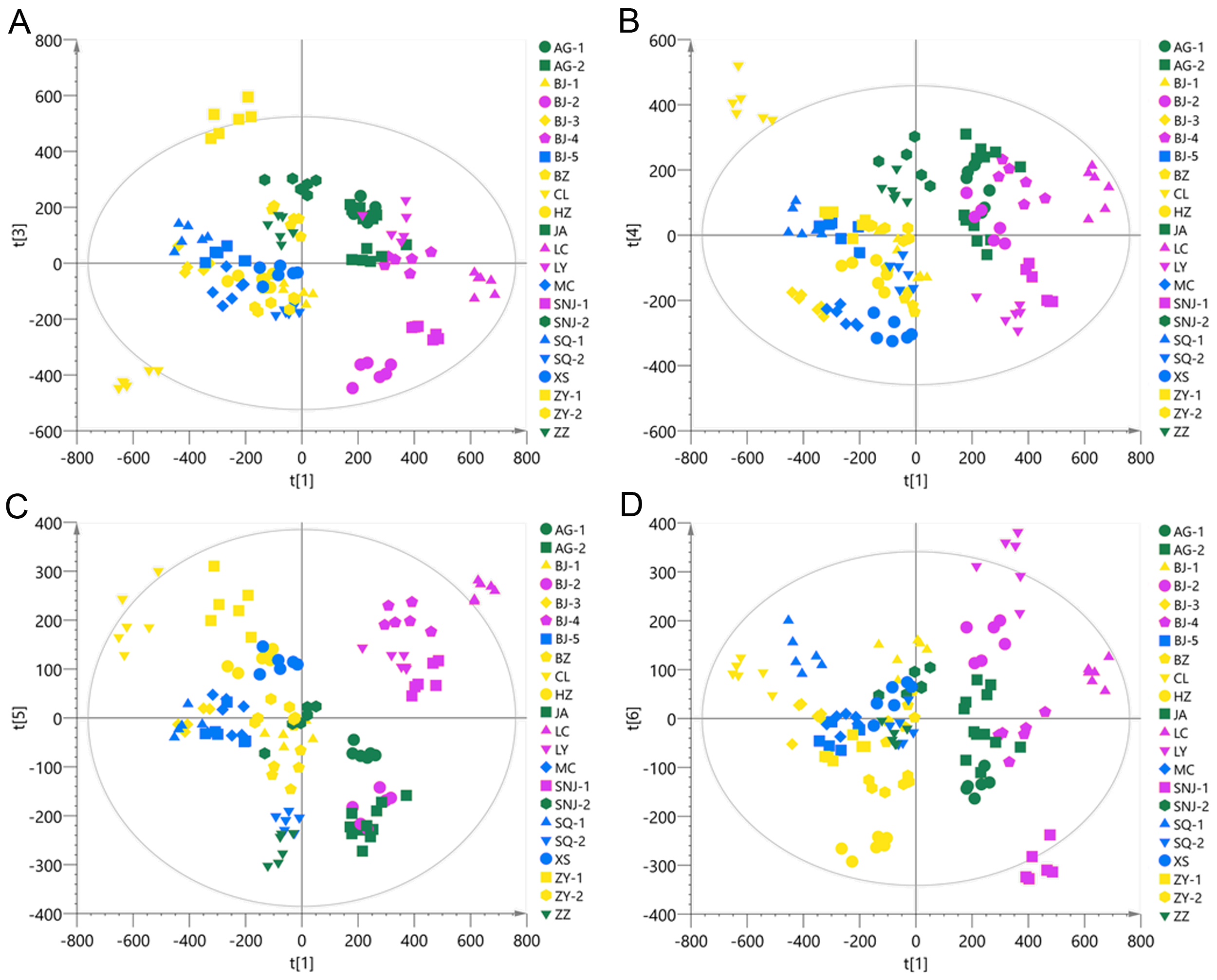

Supplement: Supplemental Information 5 — (A) PC1 pairing with PC3, PC3 accounting for 11.58% of the total variance; (B) PC1 pairing with PC4, PC4 accounting for 8.87% of the total variance; (C) PC1 pairing with PC5, PC5 accounting for 6.26% of the total variance; (D) PC1 pairing with PC6, PC6 accounting for 4.91% of the total variance. [file peerj-08-9786-s005.png]

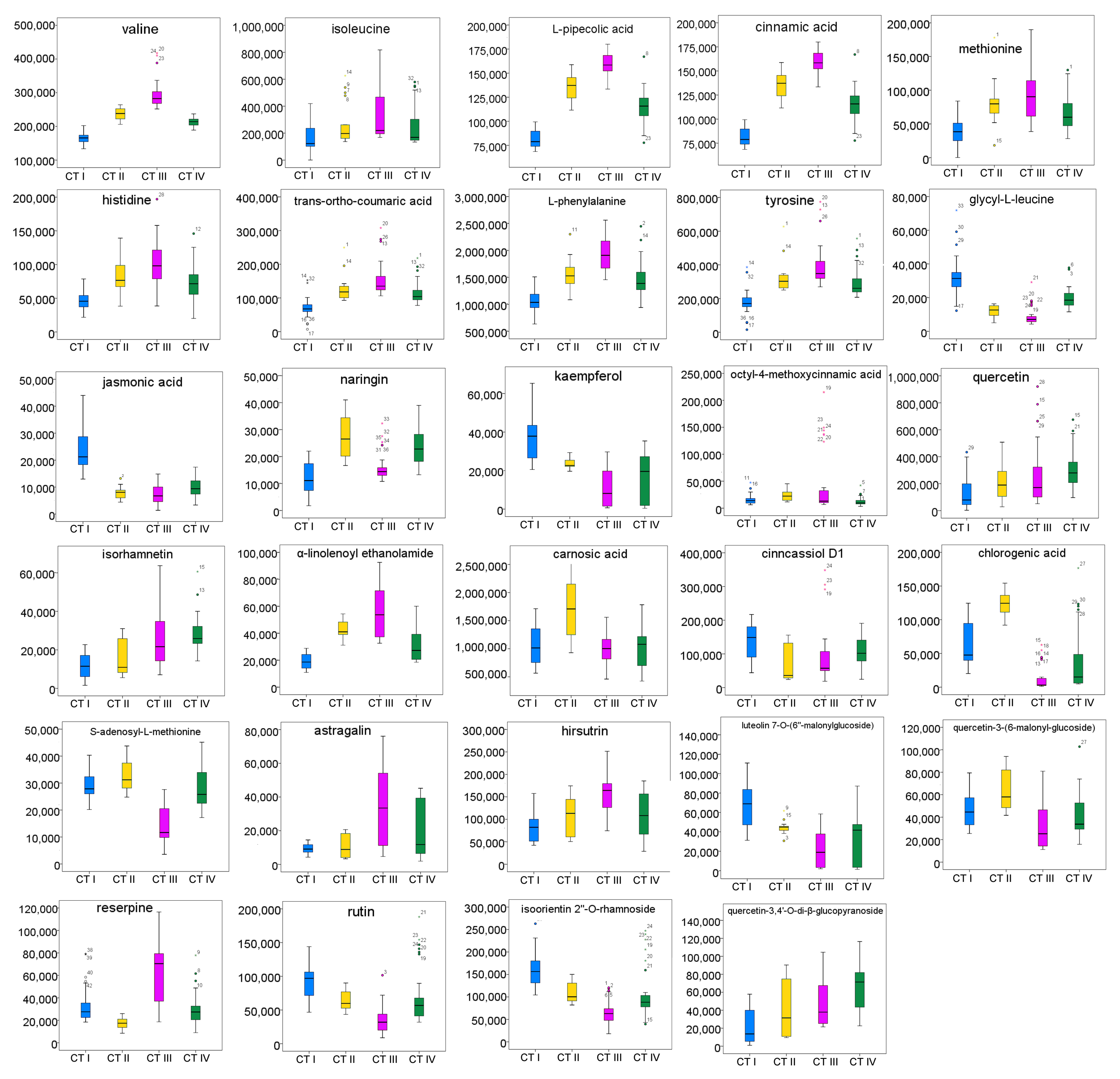

Supplement: Supplemental Information 6 — CT I,chemotype I; CT II, chemotype II; CT III, chemotype III; CT IV, chemotype IV [file peerj-08-9786-s006.png]

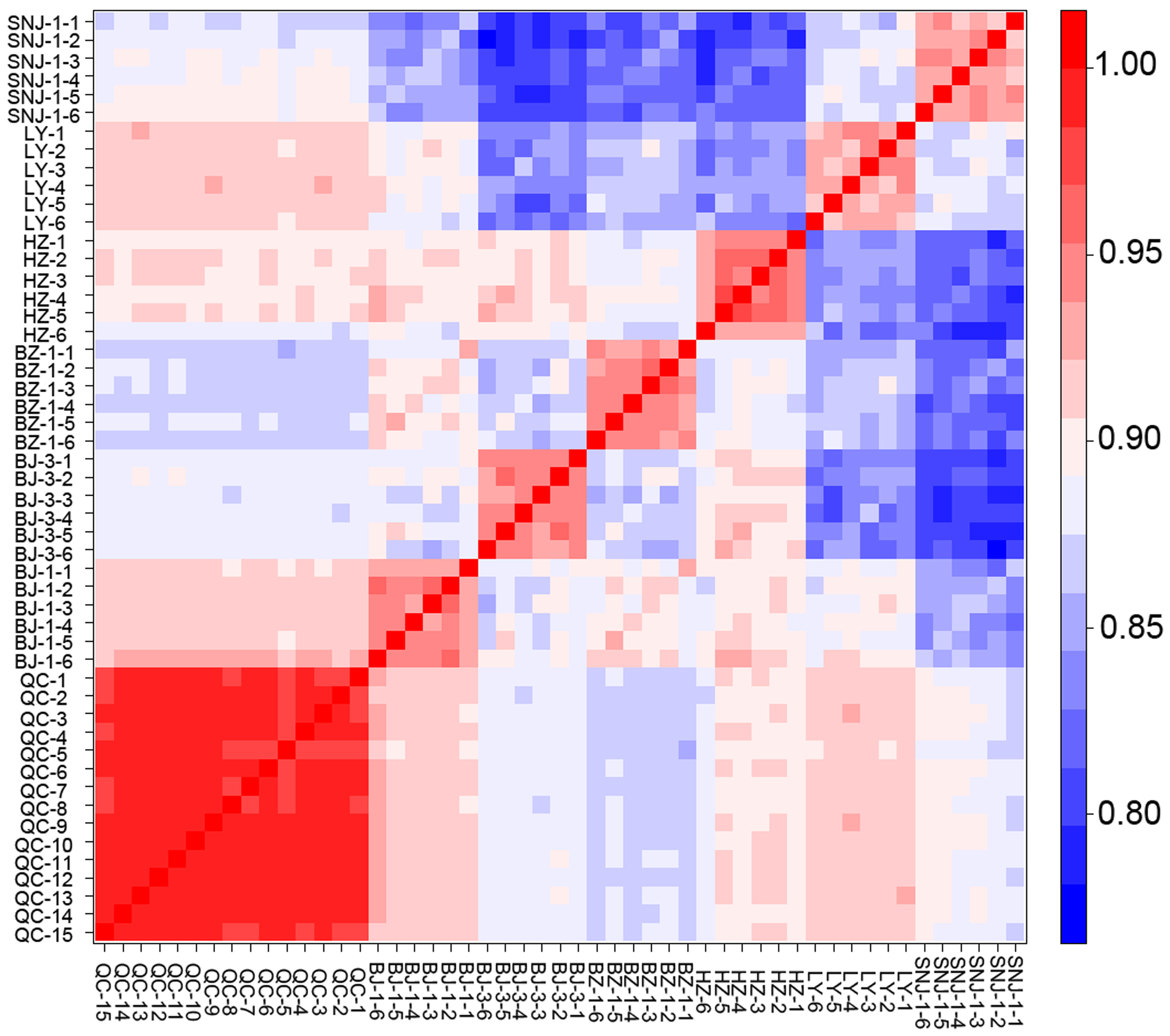

Supplement: Supplemental Information 7 [file peerj-08-9786-s007.png]
