# Supplementary material for: Chemotype classification and biomarker screening of male Eucommia ulmoides Oliv. flower core collections using UPLC-QTOF/MS-based non-targeted metabolomics
Source: PeerJ. 2020 Aug 21;8:e9786. doi: 10.7717/peerj.9786 (PMC7444510; doi:10.7717/peerj.9786)
Supplement: Supplemental Information 8 [file peerj-08-9786-s008.docx]

Table S1. Statistics of main morphological indicators describing 22 male *E. ulmoides* flower core collections

| Collection name | Geographic location of the mother tree | Branch bark color | Male inflorescence shape | Male flower color | Annual branch internode length  (cm) | Male inflorescence length  (cm) | Male inflorescence width  (cm) | Fresh weight of single male flower inflorescence (g) | Male flower length  (cm) | Male flower number of single inflorescence | Stamen length  (cm) | Stamen number of single male flower |
| --- | --- | --- | --- | --- | --- | --- | --- | --- | --- | --- | --- | --- |
| AG-1 | Anguo,Hebei | green-gray | spherical | blue-green | 14.1±4.15 | 2.02±0.18 | 1.87±0.13 | 0.76±0.12 | 1.58±0.14 | 10.33±0.58 | 1.02±0.09 | 8.60±1.35 |
| AG-2 | Anguo,Hebei | green-gray | flat spherical | blue-yellow | 15.80±5.79 | 2.29±0.21 | 2.43±0.25 | 0.79±0.18 | 1.61±0.10 | 10.33±0.58 | 1.11±0.07 | 9.53±2.03 |
| BJ-1 | Wanquanhe Road,Beijing | gray | flat spherical | blue-green | 10.40±2.17 | 2.13±0.14 | 2.10±0.17 | 1.07±0.24 | 1.43±0.11 | 10.03±1.04 | 1.01±0.07 | 8.87±1.36 |
| BJ-2 | Wanquanhe Road,Beijing | gray-brown | flat spherical | blue-green | 11.60±1.78 | 2.03±0.12 | 1.84±0.21 | 0.82±0.11 | 0.99±0.33 | 13.04±1.73 | 1.05±0.08 | 10.13±1.30 |
| BJ-3 | Tsinghua University,Beijing | green-gray | spherical | yellow-green | 19.30±8.59 | 2.02±0.25 | 1.80±0.13 | 1.37±0.24 | 1.38±0.11 | 20.33±1.53 | 1.09±0.06 | 9.60±1.40 |
| BJ-4 | *E. ulmoides* park,Beijing | green-gray | flat spherical | blue-green | 14.80±5.31 | 2.21±0.18 | 2.10±0.19 | 1.41±0.22 | 1.74±0.10 | 14.67±2.52 | 1.23±0.06 | 12.93±15.81 |
| BJ-5 | *E. ulmoides* park,Beijing | green-brown | flat spherical | yellow-purple | 8.80±1.55 | 2.35±0.20 | 2.15±0.19 | 0.86±0.14 | 1.26±0.10 | 13.02±1.06 | 1.13±0.12 | 7.33±1.29 |
| BZ | Bozhou,Anhui | green-gray | spherical | yellow-green | 14.80±5.39 | 2.24±0.12 | 2.39±0.25 | 0.95±0.29 | 1.42±0.10 | 15.67±1.53 | 1.08±0.08 | 8.40±0.99 |
| CL | Cili,Hunan | gray-brown | flat spherical | blue-yellow | 13.10±3.51 | 2.51±0.16 | 2.34±0.38 | 1.41±0.38 | 1.68±0.12 | 11.67±2.08 | 1.33±0.13 | 12.00±2.1 |
| HZ | Hangzhou,Zhejiang | green-gray | spherical | blue-green | 17.81±5.22 | 2.09±0.25 | 2.35±0.14 | 0.83±0.24 | 1.32±0.13 | 12.03±0.35 | 1.03±0.06 | 9.47±1.96 |
| JA | Ji'an,Jili | green-gray | spherical | blue-green | 16.80±7.35 | 2.17±0.25 | 2.13±0.33 | 1.58±0.57 | 1.68±0.09 | 12.02±2.65 | 1.39±0.10 | 10.20±1.21 |
| LC | Lechang,Guangdong | green-gray | spherical | yellow-green | 30.60±2.46 | 2.65±0.28 | 2.59±0.22 | 1.51±0.40 | 1.68±0.11 | 16.67±1.15 | 1.31±0.11 | 8.33±1.35 |
| LY | Lueyang,Shanxi | green-gray | flat spherical | blue-green | 15.40±2.37 | 2.52±0.26 | 1.72±0.38 | 0.91±0.20 | 1.62±0.07 | 16.01±1.73 | 1.11±0.12 | 7.73±0.96 |
| MC | Mochuan,Guangxi | green-gray | flat spherical | blue-green | 15.70±5.17 | 2.03±0.22 | 1.86±0.28 | 1.07±0.33 | 1.83±0.11 | 12.33±2.08 | 1.55±0.27 | 7.33±1.51 |
| SNJ-1 | Shennongjia,Hubei | green-brown | flat spherical | blue-green | 16.30±6.31 | 2.35±0.17 | 2.75±0.20 | 0.90±0.33 | 1.69±0.07 | 8.33±0.58 | 1.33±0.11 | 7.87±1.25 |
| SNJ-2 | Shennongjia,Hubei | yellow-brown | flat spherical | blue-green | 11.40±2.50 | 2.48±0.29 | 2.57±0.30 | 0.86±0.14 | 1.51±0.11 | 9.33±0.58 | 1.29±0.13 | 8.02±1.25 |
| SQ-1 | Shangqiu forest farm,Henan | yellow-brown | spherical | blue-green | 19.30±11.02 | 1.89±0.15 | 2.21±0.22 | 0.59±0.12 | 1.25±0.09 | 12.33±1.53 | 0.98±0.26 | 7.60±2.38 |
| SQ-2 | Shangqiu forest farm,Henan | green-gray | flat spherical | blue-green | 20.40±4.95 | 2.27±0.77 | 1.78±0.57 | 1.52±0.49 | 1.87±0.19 | 13.04±1.00 | 1.24±0.08 | 9.53±0.99 |
| XS | Xiangshui,Jiangshu | black-brown | flat spherical | blue-purple | 20.90±8.32 | 2.25±0.16 | 2.59±0.33 | 0.96±0.13 | 1.45±0.11 | 17.33±1.53 | 0.96±0.06 | 11.67±1.45 |
| ZY-1 | Zunyi forest farm,Guizhou | green-brown | spherical | blue-purple | 11.41±3.95 | 2.03±0.11 | 2.30±0.35 | 0.56±0.04 | 1.34±0.14 | 10.67±2.89 | 1.02±0.09 | 9.53±1.06 |
| ZY-2 | Zunyi forest farm,Guizhou | green-gray | flat spherical | blue-green | 11.53±2.27 | 2.56±0.31 | 2.14±0.28 | 1.12±0.26 | 1.6±0.16 | 11.67±2.89 | 1.48±0.11 | 6.87±1.30 |
| ZZ | Zhengzhou,Henan | yellow-brown | flat spherical | blue-green | 12.20±3.58 | 2.37±0.23 | 2.49±0.52 | 0.68±0.16 | 1.5±0.12 | 8.67±0.58 | 1.21±0.06 | 8.27±0.96 |
