# Supplementary material for: Chemotype classification and biomarker screening of male Eucommia ulmoides Oliv. flower core collections using UPLC-QTOF/MS-based non-targeted metabolomics
Source: PeerJ. 2020 Aug 21;8:e9786. doi: 10.7717/peerj.9786 (PMC7444510; doi:10.7717/peerj.9786)
Supplement: Supplemental Information 9 — minfrac = a minimum fraction of samples within at least one sample group, snthresh =signal to noise threshold, ppm = allowed ppm deviation of mass traces for peak picking, bw = bandwidth for grouping of peaks across separate chromatograms, mzwid= the allowed variation in the m/z dimension, mzdiff = minimum difference in m/z for two peaks to be considered as separate, profStep = the widths of profiles generated from the raw data in the m/z dimension. [file peerj-08-9786-s009.docx]

Table S2. Peak picking and alignment parameters used for XCMS

| item | parameter |
| --- | --- |
| method | centWave |
| minfrac | 0.5 |
| snthresh | 6 |
| ppm | 30 |
| peakwidth | 5,25 |
| bw | 5 |
| mzwid | 0.015 |
| mzdiff | 0.01 |
| profStep | 0.1 |

minfrac = a minimum fraction of samples within at least one sample group,

snthresh =signal to noise threshold,

ppm = allowed ppm deviation of mass traces for peak picking,

bw = bandwidth for grouping of peaks across separate chromatograms,

mzwid= the allowed variation in the m/z dimension,

mzdiff = minimum difference in m/z for two peaks to be considered as separate,

profStep = the widths of profiles generated from the raw data in the m/z dimension.
