# Supplementary material for: Chemotype classification and biomarker screening of male Eucommia ulmoides Oliv. flower core collections using UPLC-QTOF/MS-based non-targeted metabolomics
Source: PeerJ. 2020 Aug 21;8:e9786. doi: 10.7717/peerj.9786 (PMC7444510; doi:10.7717/peerj.9786)
Supplement: Supplemental Information 10 — “●”denotes that the corresponding annotated metabolite in the row where the symbol is located is the chemical marker of the corresponding chemotype in the column where the symbol is located, “○”denotes that the corresponding annotated metabolite in the row where the symbol is located is not the chemical marker of the corresponding chemotype in the column where the symbol is located [file peerj-08-9786-s010.docx]

Table S3. List of chemical markers screened by the OPLS-DA analysis for each *E.ulmoides* chemotype

| annotation NO. | MZ | RT( min) | adduct ion | formula | kegg ID | tentative identification | class | chemotype Ⅰ | chemotype Ⅱ | chemotype Ⅲ | chemotype Ⅳ |
| --- | --- | --- | --- | --- | --- | --- | --- | --- | --- | --- | --- |
| M118T59 | 118.0852 | 0.98 | [M+H]^+^ | C_5_H_11_NO_2_ | C00183 | valine | amino acids and derivatives | ● | ○ | ● | ○ |
| M132T70 | 132.1012 | 1.16 | [M+H]^+^ | C_6_H_13_NO_2_ | C00407 | isoleucine | amino acids and derivatives | ● | ○ | ● | ○ |
| M147T45 | 147.1117 | 0.75 | [M+NH_4_]^+^ | C_6_H_11_NO_2_ | C00408 | L-pipecolic acid | amino acids and derivatives | ● | ○ | ● | ○ |
| M149T145 | 149.0588 | 2.42 | [M+H]^+^ | C_9_H_8_O_2_ | C10438 | cinnamic acid | cinnamic acids and derivatives | ● | ○ | ○ | ○ |
| M150T89 | 150.0575 | 1.49 | [M+H]^+^ | C_5_H_11_NO_2_S | C00073 | methionine | amino acids and derivatives | ● | ○ | ● | ○ |
| M156T51 | 156.0759 | 0.84 | [M+H]^+^ | C_6_H_9_N_3_O_2_ | C00135 | histidine | amino acids and derivatives | ● | ○ | ● | ○ |
| M165T95 | 165.0537 | 1.58 | [M+H]^+^ | C_9_H_8_O_3_ | C12621 | trans-ortho-coumaric acid | cinnamic acids and derivatives | ● | ○ | ● | ○ |
| M166T145 | 166.0856 | 2.42 | [M+H]^+^ | C_9_H_11_NO_2_ | C00079 | L-phenylalanine | amino acids and derivatives | ● | ○ | ○ | ○ |
| M182T95 | 182.0806 | 1.58 | [M+H]^+^ | C_9_H_11_NO_3_ | C00082 | tyrosine | amino acids and derivatives | ● | ○ | ● | ○ |
| M189T119 | 189.1227 | 1.99 | [M+H]^+^ | C_8_H_16_N_2_O_3_ | C02155 | glycyl-L-leucine | amino acids and derivatives | ● | ○ | ○ | ○ |
| M211T222 | 211.1323 | 3.70 | [M+H]^+^ | C_12_H_18_O_3_ | C08491 | jasmonic acid | lipids | ● | ○ | ○ | ○ |
| M273T287 | 273.0757 | 4.78 | [M+H-C_12_H_20_O_9_]^+^ | C_27_H_32_O_14_ | C09789 | naringin | flavonoids | ● | ● | ○ | ○ |
| M287T230_1 | 287.0314 | 3.83 | [M+H]^+^ | C_15_H_10_O_6_ | C05903 | kaempferol | flavonoids | ● | ○ | ● | ○ |
| M291T360 | 291.195 | 6.01 | [M+H]^+^ | C_18_H_26_O_3_ | D05225 | octyl-4-methoxycinnamic acid | cinnamic acids and derivatives | ○ | ○ | ● | ● |
| M303T191 | 303.0499 | 3.18 | [M+H]^+^ | C_15_H_10_O_7_ | C00389 | quercetin | flavonoids | ● | ○ | ○ | ● |
| M317T202 | 317.0658 | 3.36 | [M+H]^+^ | C_16_H_12_O_7_ | C10084 | isorhamnetin | flavonoids | ○ | ○ | ○ | ● |
| M322T395 | 322.2738 | 6.58 | [M+H]^+^ | C_22_H_39_NO_2_ | C13828 | α-linolenoyl ethanolamide | organonitrogen compounds | ● | ○ | ● | ○ |
| M333T342 | 333.2038 | 5.71 | [M+H]^+^ | C_20_H_28_O_4_ | C21818 | carnosic acid | prenol lipids | ○ | ● | ○ | ○ |
| M353T316 | 353.23 | 5.27 | [M+H]^+^ | C_22_H_28_N_2_O_2_ | C17652 | cinncassiol D1 | organoheterocyclic compounds | ● | ● | ○ | ○ |
| M355T177 | 355.1023 | 2.96 | [M+H]^+^ | C_16_H_18_O_9_ | C00852 | chlorogenic acid | organooxygen compounds | ○ | ● | ● | ● |
| M399T206 | 399.1415 | 3.44 | [M+H]^+^ | C_15_H_22_N_6_O_5_S | C00019 | S-adenosyl-L-methionine | amino acids and derivatives | ○ | ○ | ● | ○ |
| M471T224 | 471.09 | 3.73 | [M+Na]^+^ | C_21_H_20_O_11_ | C12249 | astragalin | flavonoids | ○ | ○ | ● | ○ |
| M487T213 | 487.085 | 3.55 | [M+Na]^+^ | C_21_H_20_O_12_ | C05623 | hirsutrin | flavonoids | ● | ○ | ● | ○ |
| M535T230 | 535.1084 | 3.83 | [M+H]^+^ | C_24_H_22_O_14_ | C10103 | luteolin 7-O-(6''-malonylglucoside) | flavonoids | ● | ○ | ● | ○ |
| M551T203 | 551.1036 | 3.39 | [M+H]+ | C_24_H_22_O_15_ | C10103 | quercetin-3-(6-malonyl-glucoside) | flavonoids | ○ | ● | ● | ○ |
| M609T443 | 609.2711 | 7.39 | [M+H]+ | C_33_H_40_N_2_O_9_ | C06539 | reserpine | alkaloids | ○ | ● | ● | ○ |
| M611T205 | 611.1613 | 3.42 | [M+H]+ | C_27_H_30_O_16_ | C05625 | rutin | flavonoids | ● | ○ | ● | ○ |
| M617T216 | 617.1483 | 3.61 | [M+Na]^+^ | C_27_H_30_O_15_ | C03870 | isoorientin 2''-O-rhamnoside | flavonoids | ● | ○ | ● | ○ |
| M627T191 | 627.1562 | 3.18 | [M+H]^+^ | C_27_H_30_O_17_ | C12667 | quercetin-3,4'-O-di-β-glucopyranoside | flavonoids | ● | ○ | ○ | ● |

“●”denotes that the corresponding annotated metabolite in the row where the symbol is located is the chemical marker of the corresponding chemotype in the column where the symbol is located,

“○”denotes that the corresponding annotated metabolite in the row where the symbol is located is not the chemical marker of the corresponding chemotype in the column where the symbol is located.
